# Bioaccumulation and potential human health risks of metals in commercially important fishes and shellfishes from Hangzhou Bay, China

**DOI:** 10.1038/s41598-022-08471-y

**Published:** 2022-03-17

**Authors:** Md Abu Noman, Weihua Feng, Genhai Zhu, M Belal Hossain, Yue Chen, Haifeng Zhang, Jun Sun

**Affiliations:** 1 College of Marine Science and Technology, China University of Geosciences (Wuhan), No.388 Road Rumo, Wuhan, 430074 China; 2 Key Laboratory of Marine Ecosystem Dynamics and Second Institute of Oceanography, Ministry of Natural Resources, Hangzhou, 310012 China; 3 Department of Fisheries and Marine Science, Noakhali Science and Technology University, Sonapur, Noakhali Bangladesh; 4 School of Engineering and Built Environment, Griffith University, Nathan Campus, Griffith, QLD Australia

**Keywords:** Ecology, Natural hazards

## Abstract

Hangzhou Bay is facing severe anthropogenic perturbation because of its geographic position. We studied species-specific bioaccumulation of metals in commercially important fishes and shellfishes, and calculated the potential human health hazards through their consumption, which has not been reported earlier from this area. The hierarchy of metal concentration in organisms was in the decreasing order of Zn (10.32 ± 7.13) > Cu (2.40 ± 2.66) > As (0.42 ± 0.26) > Cr (0.11 ± 0.08) > Cd (0.07 ± 0.07) > Pb (0.05 ± 0.02) > Hg (0.012 ± 0.009). Except for Cd and As concentrations in fishes, metal concentrations have not exceeded the national and international guideline values. *P. laevis* and *P. trituberculatus* were the most bioaccumulative of the species studied. According to the non-carcinogenic risk assessment, children were more susceptible to metal contamination than adults. The carcinogenic risk (CR) values indicated that children were likely to experience carcinogenic threats for taking cancer-causing agents As and Cd through fish consumption. In terms of organisms, intake of two crab species, *P. trituberculatus* and *E. sinensis*, as well as the oyster species *P. laevis*, could be detrimental to consumers.

## Introduction

Heavy metal contamination in aquatic biological systems has become very common in recent years because of agrarian and mining practices and waste disposal from metallurgical and associated industries^1–3^. Due to the rapid industrial and agronomical advancement worldwide, including in China, numerous organic and inorganic contaminants have been discharged into the environment posing a significant adverse effect to biological and human life^4–6^. Owing to the center of intensive social and economic development, coastal bays and estuaries are suffering from severe disturbance^7–9^ as the sink of anthropogenic pollutants, including trace metals and metalloids^7,9–11^. As of now, major rivers, lakes, bays and reservoirs of China face several degrees of heavy metal pollution^12^. Mercury is the primary metal pollutant in Chinese waters, but copper, nickel, thallium, beryllium, and other contaminants are also severe^12^. In addition, industrial wastes, mining, metropolitan sewage, and waste created by metal smelting discharges excessive amounts of heavy metals in water bodies and seriously affecting the water quality^12^. After discharge into the waterbodies, the heavy metal can be accumulated by planktons, then aggregated in the aquatic organisms like fish, crustaceans and shellfish, and lastly, assert risks through human consumption^9,13,14^.

Aquatic organisms offer various health advantages since they possess high protein content and low saturated fats^15–18^, therefore an easily accessible source of nutrients for local inhabitants^17,19,20^. Because of their nutritional and healthful advantages, the overall fish utilisation has recently expanded a few times^21^. As an essential source of the human diet, fish quality and safety are crucial concerns for human health^6,22^. Heavy metal pollutants in the aquatic foodstuffs have become of particular interest, as they can accumulate in the diet sources from the surrounding environment^23–25^. Generally, fish can accumulate toxic elements from the contaminated water^26,27^, ingestion of suspended solids from water, ingestion of food material, adsorption through tissue or skin, and the lipophilic tissues like gills^23,28^. Some of these elements like Cu, Fe, Co and Zn are important for fish growth and metabolism^29^, but can be toxic when their concentrations increase and exceed the toxicity threshold^30^. However, non-essential elements such as Cd, As, Hg and Pb are not only poisonous to aquatic organisms but also being linked to human health problem even at low concentration^21,31^. Different metals are accumulated in fish body in various concentrations. In general, the metal concentration in live fishes follow the ranking: Fe > Zn > Pb > Cu > Cd > Hg^32^. However, many factors may impact metal uptake and accumulation like sex, age, size, reproductive cycle, swimming pattern, feeding behavior, and geographical location^33^. Besides, different affinity of metals to fish tissues, different uptake, deposition and excretion rates causes the difference of bioaccumulation in the fish body^32^. Among the different ways of metal accumulation (ingestion, inhalation, skin contact), dietary intake is the potential principle pathway of commencement to trace elements for the vast majority^23,34^. Therefore, heavy metals' uptake of long endured contaminated organisms causes severe diseases including food poisoning, liver damage, cardiovascular disorder, and even fatality^21,35^. Besides, fishes act as bio-indicators to assess the aquatic ecosystem's status as they are easily accessible in huge quantities and susceptible to accumulating trace elements^21,36^. Human health hazard assessment can also provide factual information to management authorities to take necessary steps^23^. Therefore, many local and worldwide monitoring projects have been established to evaluate the status of fish for human consumption and to assess the health of aquatic ecosystem^33,37^. Besides, there are several guidelines follows worldwide for the maximum permitted concentration of certain metals in specified foods. These national or international standards for the heavy metals in aquatic organisms serve as the scale of the degree of contamination level.

As the world's largest developing economy^38^, China has experienced a tremendous industrial bloom since 1978^39^, hence confronting severe unsettling of the estuarine and coastal environment^38^. Among China's coastal region, the health status of Hangzhou Bay (HB) is deteriorating day by day as the areas surrounding HB has undergone rapid development in the last few decades^40^. For example, the gross domestic product depicts economic growth, which has increased in this region from 197.7 billion to 1959.6 billion RMB from 1996 to 2016^39,41^. Therefore, the marine environment of HB has been deteriorated due to enhanced anthropogenic activities^30^. Besides, the HB is one of the most significant areas for the country's fisheries production^41,42^. Such as Zhoushan Islands, an islands city of HB, is China's most significant fisheries production, processing, and marketing base^43^. Hence, the assessment of ecosystems and organism’s health, and relevant human health risk is indispensable in this area. There are several studies on major and trace elements and their harmful effects, but mainly focused on either sediment or water^40,44–46^ or their transport and transformation mechanism^47^. Yet, human health risk evaluation from those heavy metals through consumption of common fishes has not been associated in the Hangzhou Bay. Although in our recently published paper^48^ we drew a brief overview of the metal contamination in water, sediment and higher trophic groups in this area, there is scanty information about the metal bioaccumulation status in common aquatic species and the consequent human health hazard.

Therefore, the aim of this research was to determine the accumulation level of heavy metals (Zn, Cu, Pb, Cd, Cr, Hg and As) in commercially important aquatic species from the Hangzhou Bay, and relevant human health risk for different age groups. Besides, this will set up a baseline information about the metal contamination level in the common fisheries species in this area.

## Materials and methods

### Study area

**Figure 1:**
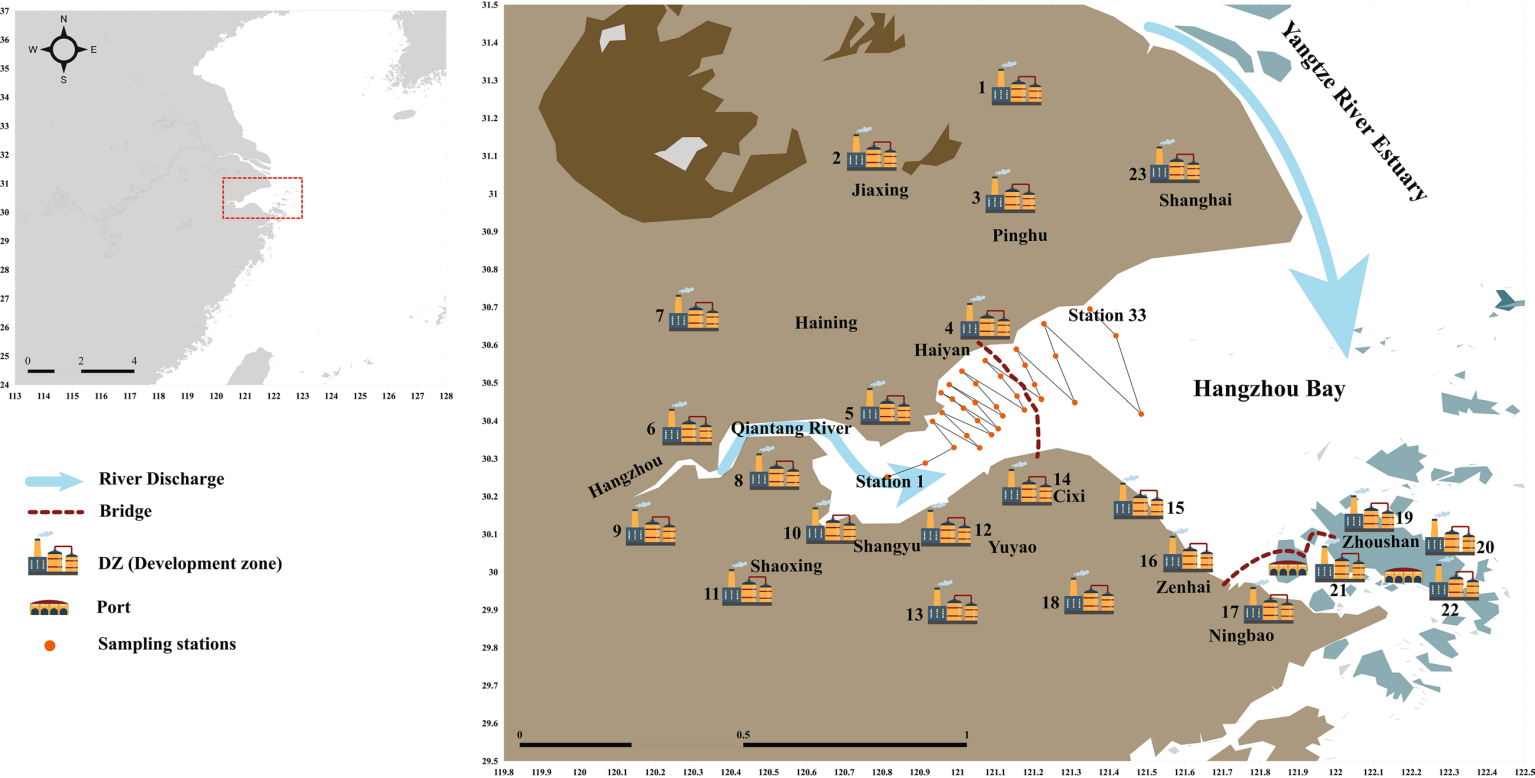
Sampling locations and the study area of the Hangzhou Bay. The development zones (DZ), bridges and ports were graphically presented following previous literature^39^.

The Hangzhou Bay, a typical funnel-shaped macrotidal estuary^47,49^, is located in the northern Zhejiang province, covering about 8500 km^2^^40^. This area is surrounded by six megacities and nine industrial parks (Fig. 1). Generally, industrials parks are the major contributors to the country's economy^38^; hence hundreds of millions of m^3^ wastewater are discharged into this area from those industries^38,50^. Moreover, except for the megacity Shanghai, there are 22 economic-technological development zones (ETDZ) and high-tech industry development zones (HIDZ) in this area (Fig. 1)^39^. Among these development zones (DZ), different areas are famous for distinguished industrial and economic activities. For example, Ningbo is a port city that handles millions of tons of cargo and ranks the world's first^39^. The logistics industry is famous in the City of Zhoushan, and the textile industry in the City of Shaoxing supports a considerable contribution to the regional economy. Besides, Shanghai city, located at the north bank of HB (Fig. 1), is one of the largest cities in the world, covering an area of 6430 km^2^ and the most prominent economic hub in China^51^. Because of this geographical setting, the HB faces severe anthropogenic disturbance and acts as the ultimate sink of pollutants from these DZ.

### Sampling parameters, methods and analysis

This report is the sequel of our recently published paper from this area^48^. Therefore, for the detailed sampling methods and analysis, the paper mentioned above is referred to. In brief, samples were collected from 33 sites during four seasons (Spring—May 2018, Summer—July 2018, Autumn—October 2018, Winter—January 2019) to analyze Cd, Cu, Pb, Zn, Hg, As and Cr in the organism's muscles. The bottom trawling collected four types of organisms, including six fish species, two crabs, two prawns, and an oyster species (Table 1). The sampled organisms were identified following existing literature^52–54^ based on their key characteristics. The edible muscles from these organisms we cut and stored in – 20 °C. All procedures strictly followed the specification of marine organism's analysis in China^55^. In brief, the muscle tissue samples were freeze-dried in the laboratory. An amount of 0.5 g samples were then placed in nitric acid (10 ml) and perchloric acid (1 ml). The solution was then put into a microwave digester for 2 h. After that for each samples the total concentrations of metals were analyzed using the Flameless Graphite Furnace Atomic Absorption Spectrometry, Flame Atomic Absorption Spectrometry and Atomic Fluorescence Spectrometry (Supplementary Table 1). Moreover, the Chinese National certified reference material (BW-HZ001 quality control sample of heavy metals in organisms) was used for the validation and accuracy. The detection level, recovery range of all metals are given in Supplementary Table 1. All the chemicals we used for this study were of analytical quality grade (Merck, Germany).

**Table 1:** Heavy metal concentrations (mg/kg) in targeted species, their feeding behavior, comparison with guideline values and related studies (values in bold denote the exceeded permissible limit).

Groups	Spcies name	N	Feeding behavior	Cu	Pb	Cd	Zn	Hg	As	Cr	References
Fish	*Coilia nasus*	34	Pelagic	0.48 ± 0.52	0.06 ± 0.06	0.02 ± 0.02	6.94 ± 3.50	0.02 ± 0.01	0.22 ± 0.10	0.09 ± 0.05	Present study
	*Collichthys lucidus*	45	Demarsal	0.36 ± 0.13	0.06 ± 0.05	0.02 ± 0.02	4.93 ± 0.69	0.01 ± 0.01	0.21 ± 0.09	0.07 ± 0.03	
	*Cynoglossus joyneri*	4	Demarsal	0.44 ± 0.04	0.06 ± 0.004	0.01 ± 0.001	6.18 ± 0.75	0.003 ± 0.0004	**0.35 ± 0.06**	0.05 ± 0.004	
	*Harpadon nehereus*	16	Benthopelagic	0.35 ± 0.17	0.07 ± 0.06	**0.06 ± 0.01**	4.80 ± 1.17	0.01 ± 0.001	0.15 ± 0.02	0.07 ± 0.04	
	*Lophiogobius ocellicauda*	7	Demarsal	0.65 ± 0.53	0.05 ± 0.02	**0.04 ± 0.01**	6.16 ± 2.14	0.01 ± 0.004	0.20 ± 0.13	0.06 ± 0.02	
	*Miichthys miiuy*	4	Demarsal	0.48 ± 0.04	0.03 ± 0.01	**0.04 ± 0.002**	4.77 ± 0.38	0.01 ± 0.005	0.19 ± 0.05	0.09 ± 0.004	
Crab	*Eriocheir sinensis*	3	Demarsal	1.14 ± 0.08	0.07 ± 0.01	0.06 ± 0.002	17.00 ± 9.48	0.02 ± 0.02	0.73 ± 0.48	0.17 ± 0.09	
	*Portunus trituberculatus*	5	Demarsal	6.62 ± 1.72	0.07 ± 0.01	0.13 ± 0.10	14.43 ± 4.18	0.01 ± 0.002	0.87 ± 0.15	0.20 ± 0.08	
Prawn	*Exopalaemon annandalei*	3	Benthopelagic	3.90 ± 3.17	0.02 ± 0.03	0.09 ± 0.01	9.98 ± 1.19	0.004 ± 0.01	0.35 ± 0.11	0.06 ± 0.01	
	*Exopalaemon carinicauda*	3	Benthopelagic	5.20 ± 3.55	0.01 ± 0.001	0.07 ± 0.06	8.89 ± 5.50	0.01 ± 0.01	0.41 ± 0.26	0.03 ± 0.03	
Oyster	*Potamocorbula laevis*	4	Demarsal	7.30 ± 4.79	0.08 ± 0.01	0.24 ± 0.29	**28.56 ± 11.85**	0.03 ± 0.03	0.72 ± 0.53	0.28 ± 0.34	
**Guideline values**											
Fish	National standard			20	2	0.6	40	0.3	–	1.5	^80^
	FAO/WHO			30	1	0.2	30	0.6	0.26		^81^
Crustacean	National standard			100	2	2	150	0.2	–	1.5	^80^
Shellfish	National standard			10	0.1	2	20	0.05	1	0.5	^80^
	FAO			30	0.5	2	30	–	–	–	^82^
**Comparison with recent reports**											
Fish	Meiliang Bay,China			0.336	0.636	0.173	–	–	–	0.118	^83^
Shellfish				1.27	1.49	0.19	–	–	–	0.60	
Fish	Pearl River, China			5.21	6.80	4.61	40.3	–	–	2.14	^72^
Fish	Taihu Lake, China			0.64	0.24	0.03	14.42	–	–	0.12	^73^
Fish	Daya Bay, China			0.9	2.2	0.011	18.9	0.23	0.10	0.36	^74^
Fish	Karnaphuli estuary, Bangladesh			12.10	13.88	0.39	–	–	4.89	3.36	^66^
Fish	Santa Maria Bay, Mexico			0.059	0.086	0.2	1.161	–	–	0.175	^76^
Fish	Palk Bay, India			0.9–8.86	0.1–0.12	0.02–0.28	18.80–55.14				^75^
Shellfish	Taihu Lake, China			1.6	0.1	1.7	127	–	1.9	–	^77^
Crustacean (Crab)	Xiangshan Bay, China			10.6	0.10	0.06	40.7	0.07	2.55	0.08	^11^
Crustacean (Prawn)				5.30	0.07	0.01	13.5	0.03	1.30	0.05	

### Bioaccumulation factor

Bioaccumulation factor (BAF) is generally calculated as the ratio between metal concentrations in muscles and water^56,57^ and considered as the degree of metal concentrations in the organism^57,58^.

$$\mathrm{BAF}=\frac{{\mathrm{C}}_{\mathrm{m}}}{{\mathrm{C}}_{\mathrm{W}}}$$

Here C_m_ is the metal concentration in the organism's muscle, C_w_ is the metal concentration in water. Further, BAF was classified as less bioaccumulative (BAF < 1000), bioaccumulative (1000 < BAF < 5000) and highly bioaccumulative (BAF > 5000)^59^.

### Human health risk assessment

#### Estimated daily intake (EDI)

EDI was assessed based on the metal concentrations in food and their daily consumption amount and calculated by the following equation:

$$EDI=\frac{C\times IR\times EF\times ED}{BW\times AT}$$

where *C* is the metal concentration in an organism's muscle (mg/kg, wet weight); *IR*- acceptable ingestion rate (0.296 kg/person/day); *EF* is the exposure frequency (365 days/year); *ED* is the exposure duration (74.8 years, which is expected average lifetime); *BW* is the average body weight (60 kg for adult, 15 kg for children); *AT* is the average exposure time for non-carcinogenic element (*EF* × *ED*)^60,61^.

#### Targeted hazard quotient (THQ)

Targeted Hazard Quotient (THQ) is the way of determining non-carcinogenic risk to the local inhabitants provided by USEPA^62,63^. THQ was calculated as the ratio of EDI and oral reference dose (*R*_*f*_*d*)^64,65^ following the equation below:

$$THQ=\frac{EDI}{{R}_{f}D}\times {10}^{-3}$$

The R_f_Dvalues for all the metals are given in Supplementary Table 2. The ratio < 1 reveals no non-carcinogenic risk effects, and the ratio > 1 implies that the community is likely to have a non-carcinogenic risk.

#### Hazard index (HI)

HI is calculated as the sum of individual non-carcinogenic risk (THQ) for all the metals (Cd, Cu, Cr, Pb. As, Hg, Zn) following the equation:

$$\mathrm{HI}=\sum_{i=k}^{\mathrm{n}}\mathrm{THQs}$$

If the HI value exceeds the threshold value (10), the exposed consumer will face significant non-carcinogenic health risk^23,66,67^.

#### Carcinogenic risk (CR)

The carcinogenic risk (CR) was assessed to evaluate the possibility of cancer in an individual over the lifetime for the exposure of cancer-causing agents^28,57,68^. The acceptable range of the carcinogenic risk is 10^–4^ to 10^–6^, and the CR values higher than 10^–4^ will probably build the likelihood of cancer-causing hazard impact^69–71^. *CR* was calculated as follows;

$$CR=\frac{\mathrm{EF}\times \mathrm{ED}\times EDI\times \mathrm{CSF}}{AT}\times {10}^{-3}$$

Here, *CSF* is the oral slope factor of cancer causing agents (mg/kg/day)^69^, which is only available for Pb (0.0085) , Cd (6.3), As (1.5) and Cr (0.5)^69^.

### Statistical analysis

The mean metal concentrations and their standard deviation (SD) in the organism's muscles were calculated using Microsoft excel. The Shapiro–Wilk tests were conducted using PAST (version 3.0) normality test, and dataset were square root transformed before further multivariate analysis. Analysis of Variance (ANOVA) and Kruskal–Wallis tests (when ANOVA did not appear) were applied with the Origin pro for the non-parametric test. Levene's test of homogeneity in terms of ANOVA was adopted to calculate the homogeneity of variance. Pearson correlation through PAST was analysed to identify the relationship between heavy metals in the organism's muscle. Principle component analysis (PCA) was implemented (by Origin pro) to show the association of heavy metals in the organism's muscles. The hierarchical cluster analysis based on Euclidean distance and the Ward-Linkage method was investigated to determine the connection between metal concentration and potential sources.

### Ethics approval and consent to participate

This study involves fish, crab, shrimp, and oyster animal testing approved by the Laboratory Animal Ethics Committee of the Second Institute of Oceanography of the Chinese Ministry of Natural Resources, and all methods were carried out in accordance with relevant guidelines and regulations.

## Results and discussion

### Metal concentrations in fishes and shellfishes

The concentrations of selected seven metals from 11 species are presented in Table 1. The mean metal concentrations (wet weight) of Cu, Pb, Zn, Cd, Hg, As and Cr in selected fishes varied as 2.40 ± 2.66, 0.05 ± 0.02, 10.32 ± 7.13, 0.07 ± 0.07, 0.012 ± 0.009, 0.42 ± 0.26 and 0.11 ± 0.08 mg/kg, respectively. The hierarchy of mean metal concentrations was Zn > Cu > As > Cr > Cd > Pb > Hg. Among different species groups, all the metal concentrations were higher in crab, prawn and oyster than the fish species but not exceeded the national and international guideline values. Some species of fish exceeded the guideline values of Cd and As concentrations (Table 1).

Overall the concentrations of Zn were highest compared to all metals in all species. The maximum concentrations of Zn were recovered from *P laevis*. They maintained the following decreasing trend: *P. laevis* > *E. sinensis* > *P. trituberculatus* > *E. annandalei* > *E. carinicauda* > *C. nasus* > *C. joyneri* > *L. ocellicauda* > *C. lucidus* > *H. nehereus* > *M. miiuy* (Table 1). In our study, the concentrations of Zn ranged from 2.66 to 41.5 mg/kg (mean 7.15 mg/kg), and the average concentration of Zn was 4.3, 187, 133, 860, 24 and 93 folds higher than the average concentrations of Cu, Pb, Cd, Hg, As and Cr respectively. The mean concentrations of Zn in fish, crab and prawn were within the national and FAO/WHO guideline values. But the Zn concentrations in oyster exceeded the national first limit standard. The concentrations of Zn in oysters varied from 41.5 to 16.52 mg/kg (average 28.56 ± 11.85 mg/kg). Besides, the Zn concentrations in all fish species were lower than the related studies at the Pearl River^72^, the Taihu Lake^73^, the Daya Bay^74^ and the Palk Bay^75^; but higher than the concentration recovered from the Santa Maria Bay, Mexico^76^. Similarly, in crabs, prawns and oysters, the Zn concentrations were lower than the previous study at the Xiangshan Bay^11^ and the Taihu Lake^77^ (Table 1). Invertebrates like oysters and barnacles accumulate specific metals (Cu and Zn) to phenomenally high concentrations^78^. Along with the metal characteristics and organisms bioaccumulation capability, the various environmental processes might be essential to regulate the bioavailability^79^. Such as, significant correlations were observed between the sediment and oysters heavy metal concentrations^78^. In the previous report, it was discerned that most of the heavy metal concentrations in the sediment of this area were within the national and international standard^48^. Besides, high metal concentrations are sometimes found in the sediment but not in the oyster’s tissue^79^. The high particulate matter in the sediment and body size of the oysters may cause a tremendous difference between the metal concentrations^78,79^. Moreover, a high amount of metal can be found in the gills, mantle and viscera^78^, which was not focused in this study.

According to the national guideline value of China, the permissible limit for Cu concentrations is 20, 100 and 10 mg/kg, respectively, in fish, crustaceans and shellfish. FAO assigned that 30 mg/kg is the permissible limit of Cu concentrations for fish and shellfish^82^. The concentrations of Cu in our study varied from 0.07 to 14 mg/kg (mean 2.40 mg/kg) among all species. The maximum concentrations of Cu recovered from the collected oyster species *P. laevis.* Among the fish species, the maximum concentration of Cu found in *L. ocellicauda* and minimum in *H. nehereus* (Table 1)*.* Comparing with the fish species, crab, prawn and oyster comprised considerably higher Cu concentrations. Cu is a vital element for the body as it forms hemoglobin and other essential enzymes, but excess Cu consumption is responsible for the malfunction of the liver and kidney^23,84,85^. However, in our study, none of the species contained a higher concentration of Cu than the national and international guideline values. The mean Cu concentrations in fishes (0.45 mg/kg) were higher than the concentrations recovered from the Meiliang bay^83^ and Santa Maria Bay^76^ but lower than most of the other related studies^66,72–75^. However, the concentrations of Cu in crustaceans was lower than the reported value in the Xiangshan Bay^11^, whereas, in oysters it was higher than the previous report^77,83^ (Table 1).

In comparison with Zn and Cu, the concentrations of Pb, Cd, Hg and Cr concentrations were very lower in all the species. Among them, the concentrations of Pb ranged from 0.006 to 0.3 mg/kg (mean 0.059 mg/kg); and the maximum concentrations of Pb found in *P. laevis* (mean 0.08 ± 0.01 mg/kg) and minimum in *E. carinicauda* (0.01 ± 0.001 mg/kg) (Table 1)*.* The national permissible limit for Pb concentrations is 2, 2 and 0.1, respectively, for fish, crustaceans and shellfish. FAO set up the guideline values as 1 and 0.5 mg/kg for fish and shellfish, respectively. Therefore, the Pb concentrations in our study were not only within the guideline values, but also lower than the national and international reports^6,11,66,72,74–77,85^. The concentrations of Hg did not vary largely among the tested species, though the maximum concentrations recovered from *P. laevis.* Similar to other metals, the concentration of Cr was maximum in *P. laevis* followed by *P. trituberculatus* and *E. sinensis.* Both of the elements' concentrations were within the national and international standard values set up by FAO^81,82^ (Table 1).

Cd concentration was maximum in *P. laevis and* minimum in *C. joyneri.* According to the Chinese national standard, the guideline value for Cu concentration in fish is 0.06 mg/kg, 2 mg/kg for both crustacean and shellfish. According to FAO, the permissible limit for Cd concentrations is 0.2 and 2 mg/kg for fish and shellfish. In our study, *H. nehereus, L. ocellicauda* and *M. miiuy* crossed the FAO permissible limit, whereas other species were in line with the standard criteria. The mean Cd concentration in fish (0.023 mg/kg) was almost similar to the values reported from the Taihu lake (0.03 mg/kg)^73^, higher than the Daya bay (0.011); but much lower than Meiliang Bay (0.173 mg/kg)^83^, Pearl River (4.61 g/kg)^72^, and Karnafuli estuary (0.39 mg/kg)^66^. Besides, the Cd concentrations in shellfish exceeded the value reported earlier from the Meiliang bay (0.19 mg/kg)^83^ but were lower than the Taihu lake (1.7 mg/kg)^77^. In terms of crustacean, both crab species, especially *P. trituberculatus* had higher concentrations of Cd than the similar study in the Xiangshan bay^11^; and both of the prawn species had higher Cd concentrations of that report (Table 1).

In terms of As, the concentrations ranged from 0.04 to 1.48 mg/kg (mean 0.42 ± 0.26 mg/kg), and the mean concentration was higher in crab and prawn than in other groups. *P. trituberculatus* comprised the maximum As concentrations, and the hierarchy of spices in terms of As concentration was as follows: *P. trituberculatus* > *E. sinensis* > *P. laevis* > *E. carinicauda* > *E. annandalei* > *C. joyneri* > *C. nasus* > *C. lucidus* > *L. ocellicauda* > *M. miiyu* > *H. nehereus.* FAO set up the maximum permissible limit of As is 0.26 mg/kg in fish and 1 mg/kg in shellfish^81,82^. In our study, *C. joyneri* exceeded the permissible limit of As concentration, but other species was within the permissible limit. The inorganic As is more deadly than the organic form and can act as a cancer-causing agent for human beings if they consume the inorganic arsenic for a longer period^84^. But, it is a matter of satisfaction that most of the As concentrations in seafood are in organic form and can be directly excreted through the urine^66,84,86^. Our finding is comparable with the similar study in the bay and coastal region in China and worldwide (Table 1). The mean As concentration in Daya Bay was reported as 0.10 mg/kg in fish^74^, which is lower than our study. In Karnafuli estuary Bangladesh, the reported As concentration was 4.89 mg/kg in fish^66^, and in our study, the concentration was very lower than their findings. Besides, the concentration of As in shellfish, crab and prawn in Taihu lake^77^ and Xiangshan Bay^11^ were higher than the concentrations recorded in our study (Table 1).

Three species (*C. nasus*, *C. lucidus*, *P. laevis*) were sampled in four seasons for comparing the seasonal variation of heavy metal concentration. On their muscles, the concentrations of Zn were higher than other metals throughout the year. In *C. nasus*, the concentrations of Zn were maximum in summer (11.84 mg/kg) followed by spring (8.66 mg/kg), autumn (4.8 mg/kg) and winter (3.50 mg/kg). Cu concentrations were maximum in spring (1.09 mg/kg) followed by winter (0.47 mg/kg), summer (0.32 mg/kg) and autumn (0.21 m/kg) (Supplementary Fig. 1). As concentration in *C. nasus* followed the trend of spring (0.38 mg/kg) > autumn (0.21 mg/kg) > summer (0.20 mg/kg) > winter (0.12 mg/kg). Pb concentrations were higher in summer (0.117 mg/kg) and spring (0.114 mg/kg) whereas Cr concentrations were higher in summer (0.13 mg/kg) and autumn (0.09 mg/kg). Overall the mean metal concentrations in *C. nasus* were organised in four seasons as: summer > spring > autumn > winter. In *C. lucidus.* Zn concentrations relatively higher in winter (5.5 mg/kg) and summer (5.24 mg/kg), and lower in spring (4.67 mg/kg) and autumn (4.3 mg/kg) (Supplementary Fig. 1). Cu concentrations in *C. lucidus* were higher and almost similar in winter (0.46 mg/kg) and spring (0.43 mg/kg) and lower in summer (0.27 mg/kg) and autumn (0.25 mg/kg). In terms of As, the concentrations on the *C. lucidus,* in spring (0.3 mg/kg) the concentrations were maximum followed by summer (0.21 mg/kg), winter (0.19 mg/kg) and autumn (0.12 mg/kg). The hierarchy of mean metal concentrations in *C. lucidus* in four seasons was winter > summer > spring > autumn. In the tested oyster species, *P. laevis*, Zn and Cu concentrations were higher in four seasons than other metals. In spring (41.5 mg/kg) and summer (35.47 mg/kg), the Zn concentrations were higher than autumn (20.74 mg/kg) and winter (16.52 mg/kg) (Supplementary Fig. 1). But, the concentrations of Cu were maximum in autumn (14.03 mg/kg), followed by spring, summer and winter. The As concentrations were considerably higher in autumn (1.48 mg/kg) than in the other three seasons. Other metal concentrations not varied largely on *P. laevis.* As a result, the average metal concentrations in *P. laevis* maintained the seasonality as follows: spring > summer > autumn > winter. The findings of our study are comparable with similar regional studies on seasonal pollution in the Meiliang Bay of Taihu Lake^6^. They reported that higher concentrations of metals were found in summer and winter, but both the fish and oysters showed great seasonality in terms of metal types^6^. In fact, various species take up various types of metals from both water and foodstuffs, and their physical properties are additionally influenced by ingestion and filtration rates, food quality, physiological states and ecological variables^87,88^. Therefore, it is not astounding that the seasonality of metals in different species was unique, regardless of having been sampled in the same period^87^.

In our study, the demersal species comprised the highest metal concentrations, followed by benthopelagic and pelagic species. Demersal species lives close to the bottom^89^, and many of them possess special features (such as modified fins for fish) to crawl over the bottom (crab, oyster). Those species are bottom feeders, mostly carnivorous and primarily feed on fish, benthic macroinvertebrates and zooplankton, in contrast with the pelagic fishes. Besides, the benthopelagic fishes inhibit just above the bottom and mainly feed on benthos or zooplankton^90^. Therefore, the demersal fishes had a higher metal concentration as they inhibit near the bottom^66^.

### Source identification of metals

Two-way ANOVA revealed that most metal concentrations were significantly different among the sites and seasons at a 95% confidence level (P < 0.05). In contrast to these findings, Cr concentrations in fish muscles were not significantly different among the sites and seasons. Similarly, the concentrations of Pb and As were not significant among the sites, and Cd was not significantly different among the seasons. One-way ANOVA tested the seasonality of metal concentrations, and all metals in *C. nasus* and *C. lucidus* showed significant variation in four different seasons (Supplementary Fig. 1). The Kruskal- Walis test was implemented for *P. laevis* as the sample size was smaller. It revealed a significant difference in metal concentrations among the seasons (P < 0.01). Levene's test of ANOVA revealed that the metals were not homogenously distributed among the sites and seasons.

As Hangzhou Bay is a complex nature of ecosystems, we have applied both univariate and multivarite approaches i.e., pearson correlation, principal component analysis (PCA) and cluster analysis (CA) to identify the sources of metals. Pearson correlation analysis of metal concentrations in organism's muscles is shown graphically in Supplementary Fig. 2. According to the correlation analysis, strong positive relationships were found between the metals in the organism's muscle. Such correlations between particular elements in nature may depict a similar degree of contamination and discharge from a similar source of contamination^23,91^. Among them, the strongest relationships were found between Cu–Zn (r = 0.73), Cu–As (r = 0.72), Cu–Cd (r = 0.87) at 95% confidence level. Zn, As, Cr and Cd showed significant positive relationships with all other metals, but Pb did not show any correlations with other metals (Supplementary Fig. 2). Therefore, this correlation reveals that most of the metals which are significantly correlated with each other may originate from the same source, either anthropogenic or natural, whereas Pb might be released from different source.

**Figure 2:**
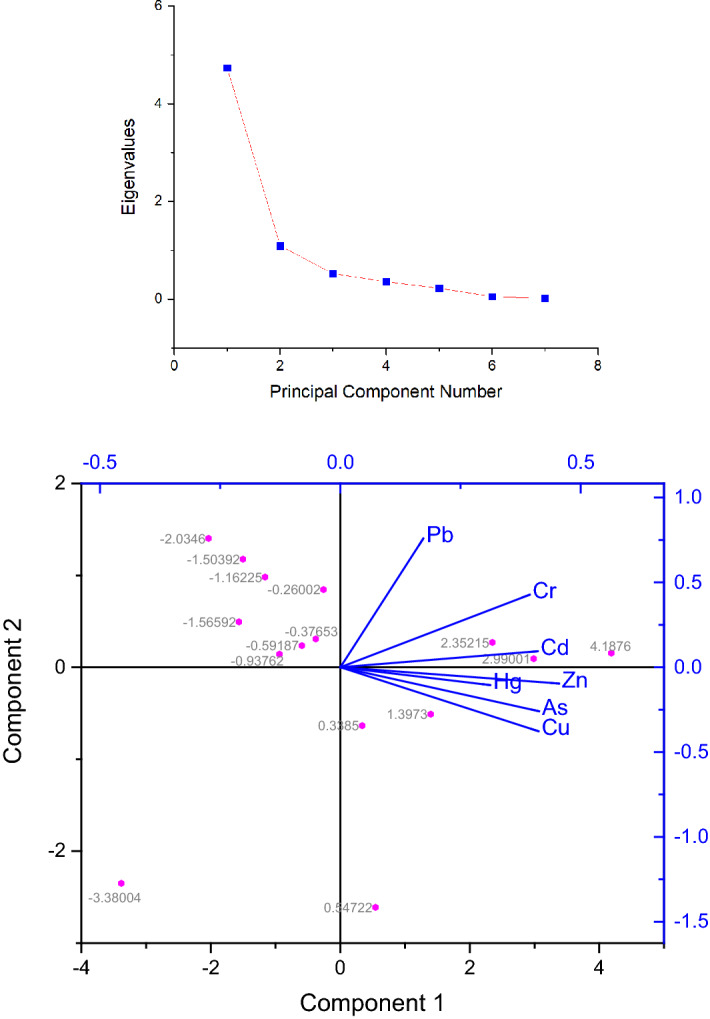
Scree plot and principal component analysis (PCA) loadings plot of seven heavy metals in organism’s samples.

Among the multivariate analyses, PCA is based on eigenvalue analysis of the correlation matrix which estimates the correlation structure of the variables in a multidimensional data set^92^. Each variable has a loading which shows how much each variable contributes to the meaningful variation in the data and to interpret variables relationship. Practically, higher number PC components that explain only a small proportion of variance is ignored^93^. In this study, the association of metals in organisms’ muscles were interpreted through PCA bi-plot (Fig. 2). The first two components of the PCA have explained 77.54% of total variation in metal concentrations and the rest components showed small amount of variation. PC1 was dominated by Cu, Zn, Cd and As with the loadings of 0.41, 0.45, 0.41 and 0.41, respectively. On the other hand, PC2 was highly associated with Pb (loading 0.75) and moderately with Cr (loading 0.42). In addition, partial representation was found for Hg in PC1 (loading 0. 37) and PC3 (loading 0.71). Therefore, analysis of the PCA results interprets that Cu, Zn, Cd and As were originated from similar source i.e. anthropogenic origin as their levels exceed (in some cases) or close to the legal limits, and on the other hand, Pb being the far below of the permissible limit was originated from natural source. Again partial association of Hg in two PCs suggests mixed sources of origin.

**Figure 3:**
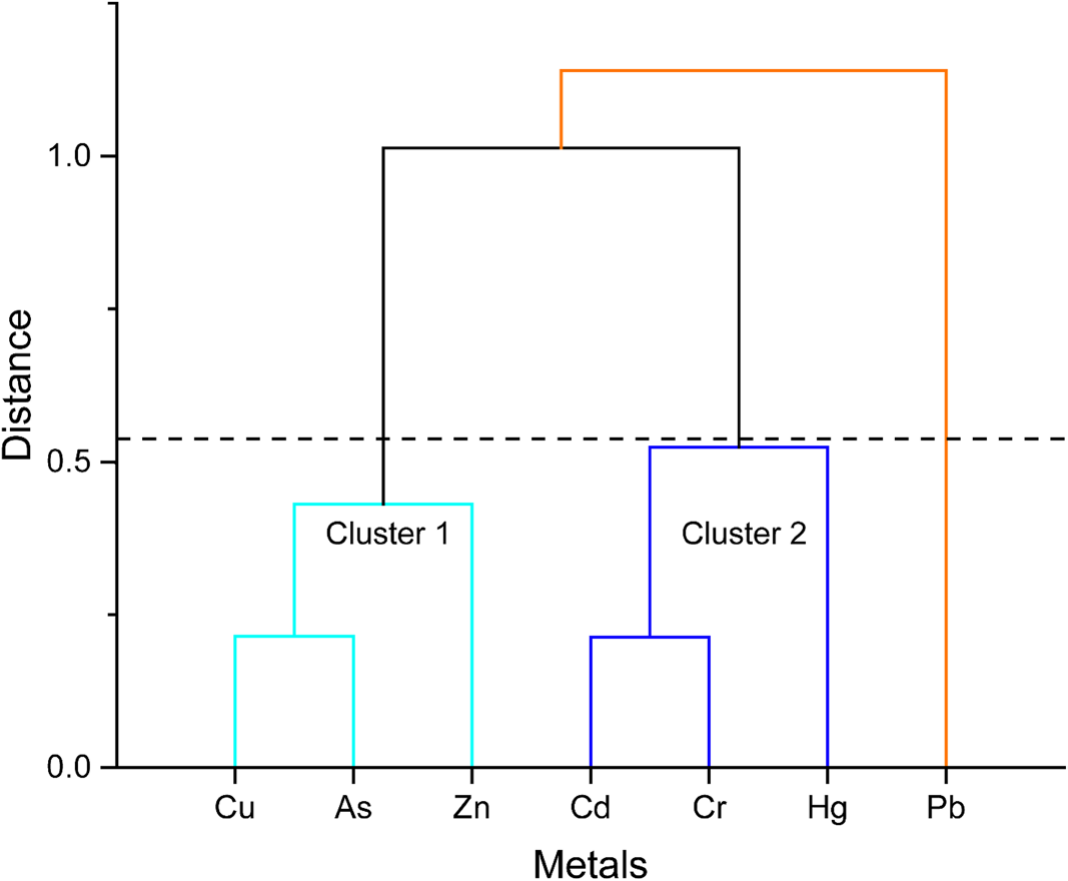
Hierarchical cluster analysis based on the Euclidean distance of the metals in selected species.

Besides, associations for interdependence among the different metals in the study area were also established by means of the hierarchical cluster analysis based on Euclidean distance. The results demonstrated that among the seven metals, six metals (Cu, As, and Zn in cluster 1; Cd, Cr and Hg in cluster 2) were grouped in two major clusters whereas, Pb showed a clear distinction forming an individual group. This findings also indicated that most of the metals were originated from the same sources excepting Pb. It is reported earlier that the heavy metals in this area originates from similar sources^40,94^ and are related to anthropogenic activities^40^. Besides, the transport of terrigenous clastic particles was an unequivocal definitive of the formation and dissemination. At the same time, the transition mechanism of trace elements of various substrates and contamination of marine aquaculture were likewise significant contributors to trace metals' sources in this region^94^ (Fig. 3).

### Bioaccumulation factor (BAF) of aquatic species

**Figure 4:**
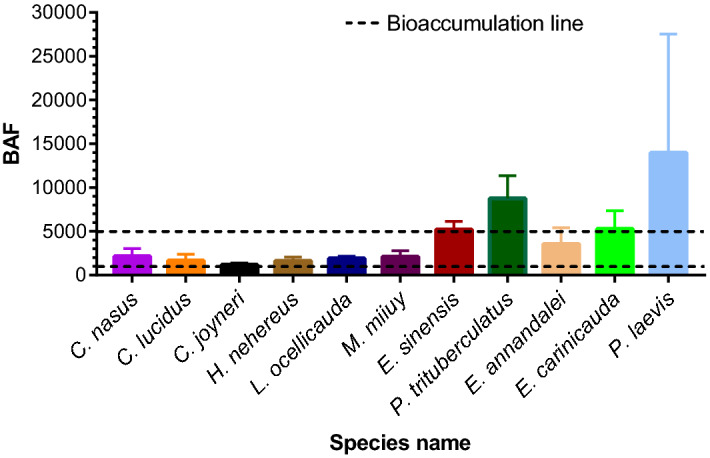
Bioaccumulation factor (BAF) of the selected species from the Hangzhou bay (Dashed line indicates the Bioaccumulation line).

The BAFs of the heavy metals in selected organism's muscles are graphically represented in Fig. 4. BAFs associate the number recovered from the portion of collected concentration in a specific organ of a species and the habitat quickly and effectively^23,85,95^. In our study, the highest mean BAF was encountered for Cu, lowest for Cr; and followed the decreasing trend of Cu (1640) > Zn (736) > Hg (387) > Cd (366) > As (288) > Pb (173) > Cr (82) in the organism's muscle. Actually, Cu is effectively persistent in an organism's muscles due to being an essential component of living tissue^66,96,97^. In terms of organisms, all species were bioaccumulative, but *P. trituberculatus* and *P. laevis* were extremely bioaccumulative. Among the selected species, the oyster, crab and prawns were more bioaccumulative than the fishes. Among all the species, the BAF value was maximum in *P. laevis* (13998) and minimum in *C. joyneri* (1098) (Fig. 4). In fact, the bioaccumulation of an aquatic species relies on its characteristics, metabolism of the inspected tissue, invasion pathways, and habitat condition^66,98^. Therefore, each species has a specific bioaccumulative receptivity regarding various metals^23,69,99,100^.

### Potential human health risk assessment

#### Non-carcinogenic risk (EDI, THQ and HI)

Heavy metals poisonous quality could incline people to health hazards through consumption of defiled aquatic food; therefore, it is crucial for assessment^87^. Based on the oral reference dose *R*_*f*_*D*, the EDI was calculated to measure both the non-carcinogenic and carcinogenic risk of metal consumption through seafood^101^. Besides, the EDI value denotes the exposure of heavy metals, which is performed to evade any detrimental impact on human health^102^. The hazard analysis was conducted for two age groups, children and adults. The calculated EDI of the examined metals associated with fish, crab, prawn and oyster are presented in Supplementary Table 2. For both groups, the EDI of metals through the organism muscles were organised as follows Zn > Cu > As > Cr > Cd > Pb > Hg (Supplementary Table 2). The EDI was higher for the children than the adult, especially for children the EDI value of As, Cd and Zn; and for adults only As had higher EDI values than the Recommended Daily Allowance (RDA) provided by WHO^103^ (Supplementary Table 2).Therefore, the higher EDI than the RDA guidelines, revealed that here is a possibility of higher health impact associated with As, Cd and Zn to the consumers, mostly children would be more susceptible.

**Figure 5:**
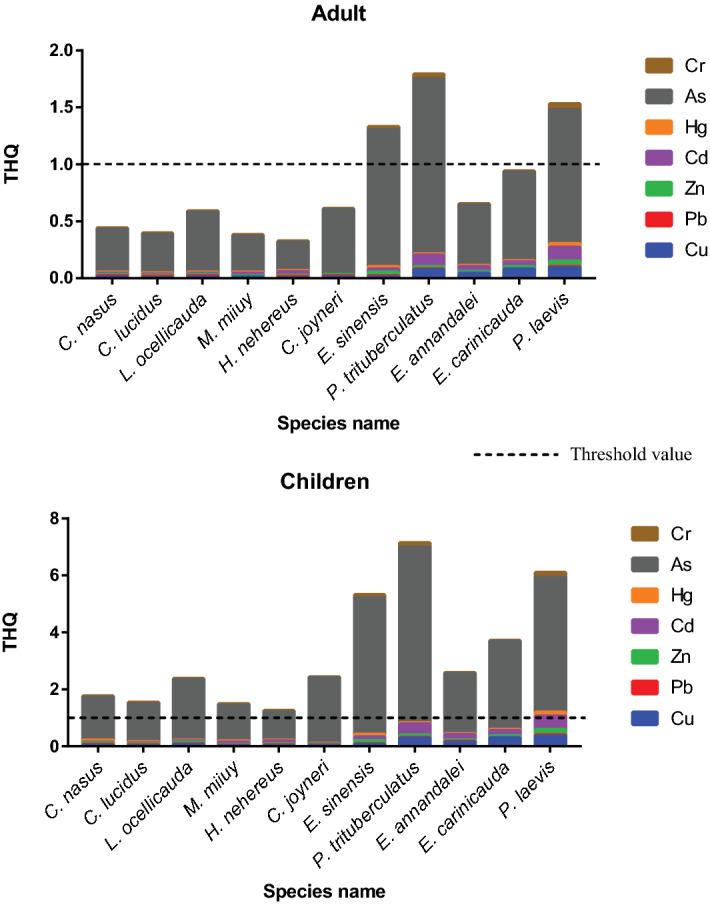
Targeted hazard quotient (THQ) for the children and adult consumer of targeted species.

The estimated THQ values (Fig. 5) were higher in the children group than the adult. Particularly the THQ of As was significantly higher in both groups than other metals. For the adult people, the THQ of As for the consumption of *E. sinensis, P. trituberculatus,* and *P. laevis* exceeded the threshold value (> 1) suggested by USEPA^104^. Whereas, for the children, the THQ of As was higher (> 1) for all the species but maximum in three species similar to the adult. For both groups, except for As, all the other metals in all the species possessed lower THQ values, which were far lower than the threshold value (< 1). The accumulated THQ of all metals in the concern of HI, also much higher for the children. Though the HI was within the threshold limit (both for adults and children) for all the organisms, it was much higher for oysters, prawns and crabs (Fig. 5). Therefore, the local consumer can suffer from As contamination for the long term seafood consumption. Moreover, an organism’s age and size could be important factors for metal accumulation and health risks^28,105^. Though no definite trend of metal accumulation was observed in *Capoeta umbla* fish species, higher concentrations were found in medium-aged fish compared to the small-sized or older fish^106^. However, metal concentration is determined by the organism’s feeding rate with the developmental stage rather than age^105^. Besides, the larger size organisms tend to accumulate more metals^105^; consequently, they may pose more threat to human health. A previous study reported that the larger-sized *Labeo rohita* accumulated more metals and had higher hazard potential than smaller fishes like *Glossogobius giuris*, *Puntius sophore* and *Puntius chola*. Our study did not focus on size-based metal accumulation; hence all the fishes sampled were within 200–230 gm and posed little threat to humans (low THQ). However, our results emphasize that the organism’s trophic guild and habitat preferences play significant role in the metal accumulation and consequent health hazard. Many earlier studies reported that the contamination level differ largely on the food habitat of the organisms^107–109^. Therefore, though the two crab species *P. trituberculatus* and *E. sinensis* weighed lower than the fish (200 ± 40 g and 158 ± 42 g, respectively), they accumulated higher metals and hazard potential. Similarly, the oyster species *P. laevis* was weighed 178 ± 70 g, also showed high bioaccumulation capacity and THQ value for both adults and children, whereas fishes showed low THQ values although weighed more. But, suppose the organisms sampled from the same habitat type and feeding guild, then the organism’s size is an important factor regarding metal accumulation and the hazard potential to their consumers. However, the THQ and HI have no particular dose relationship that's why they are not considered as an immediate estimation of hazard concern^110^.

#### Carcinogenic risk (CR)

The estimated CR of several elements (Pb, Cd, As, Cr) for adults and children are addressed in Table 2. CR values lower than 10^–6^ denote the metals' negligible exposure, whereas 10^–6^ to 10^–4^ means the acceptable range, and higher than 10^–4^ indicates the terrible exposure^84,111^. For the adults, all the metals possessed the CR values within the limit of 10^–4^–10^–6^, though the As showed high carcinogenic risk than other elements. In line with the previous studies, children were more susceptible for CR exposure^23,101,112^. For the children, both the As and Cd showed high CR values than the permissible limit for several species. As showed terrible CR exposure for the children through the consumption *C. joyneri, E. sinensis, P. trituberculatus, E. carinicauda* and *P. laevis.* Whereas, besides those species, *E. annandalei* showed terrible CR exposure for children in terms of Cd (Table 2). For both groups, CR values of Pb were negligible, and the metal exposure for the seafood consumption was organised as As > Cd > Cr > Pb. Therefore, the local consumers are at carcinogenic risk associate with the toxic As, and for children, Cd is an additional matter of carcinogenic concern. It was reported in the Persian Gulf that carcinogenic risk in terms of As could be detrimental to local consumers, though it did not show any non-carcinogenic risk. Actually, 90% of the carcinogenic risk has been found in the As polluted seafood^66^. However, the inorganic form of As is more deadly than the organic form^57,113^, and around 90% of the aggregated As can be evaluated as organic form^84^. Chronic exposure of inorganic As to human lead to various malfunctions^114^ including the organ failure such as respiratory tract, circulatory system, digestive system, nervous system and liver^115^. Besides, Cd is also responsible for endocrine malfunctioning, which can cause the failure of the essential organ such as the kidney and brain^116^. Moreover, long term contamination of Cd may cause the dysfunction of the blood circulatory system^117^, bone softening and prostate cancer^118^. Therefore, the USEPA categorized Cd as a priority contaminant and considered as "carcinogenic to humans" (Carcinogenic classification–B)^23^. However, the tissue samples in this study were digested by nitric acid and perchloric acid system, and total heavy metal concentrations were used in the health risk assessment. The use of total heavy metal in health risk assessment is seen to overestimate the risks calculated and cause uncertainties^119^. Besides, the total metal consumption depends on the gross quantity of dietary intake besides the aquatic foods and their metal concentration. Therefore, the health risk of heavy metal should be considered the bioavailability of metal concentration and the total dietary intake along with seafood in future.

**Table 2:** Human carcinogenic risks (CR) through the consumption of targeted species. (Values in bold denote the terrible CR exposure).

	Spcies name	CR (adult)				CR (child)			
		Pb	Cd	As	Cr	Pb	Cd	As	Cr
Fish	*Coilia nasus*	2.83E−07	7.04E−05	1.69E−04	2.23E−05	1.13E−06	2.81E−04	6.76E−04	8.93E−05
	*Collichthys lucidus*	2.76E−07	6.16E−05	1.51E−04	1.75E−05	1.10E−06	2.46E−04	6.03E−04	6.99E−05
	*Lophiogobius ocellicauda*	2.75E−07	7.78E−05	2.40E−04	1.39E−05	1.10E−06	3.11E−04	9.59E−04	5.54E−05
	*Miichthys miiuy*	1.08E−07	1.19E−04	1.42E−04	2.23E−05	4.32E−07	4.75E−04	5.69E−04	8.90E−05
	*Cynoglossus joyneri*	2.64E−07	2.89E−05	2.58E−04	1.13E−05	1.06E−06	1.15E−04	**1.03E−03**	4.53E−05
	*Harpadon nehereus*	2.65E−07	1.77E−04	1.13E−04	1.46E−05	1.06E−06	7.09E−04	4.50E−04	5.83E−05
Crab	*Eriocheir sinensis*	2.80E−07	1.97E−04	5.40E−04	4.30E−05	1.12E−06	7.89E−04	**2.16E−03**	1.72E−04
	*Portunus trituberculatus*	2.73E−07	6.16E−04	6.94E−04	6.16E−05	1.09E−06	**2.47E−03**	**2.78E−03**	2.47E−04
Prawn	*Exopalaemon annandalei*	1.29E−07	2.77E−04	2.38E−04	1.49E−05	5.16E−07	**1.11E−03**	9.51E−04	5.95E−05
	*Exopalaemon carinicauda*	3.35E−08	2.77E−04	3.50E−04	1.04E−05	1.34E−07	**1.11E−03**	**1.40E−03**	4.14E−05
Oyster	*Potamocorbula laevis*	3.50E−07	7.41E−04	5.31E−04	6.86E−05	1.40E−06	**2.96E−03**	**2.13E−03**	2.74E−04

## Conclusions

The concentrations of seven heavy metals in the 11 aquatic organism's muscles were analysed from the HB area. Overall, the hierarchy of mean metal concentrations was Zn > Cu > As > Cr > Cd > Pb > Hg. Among different species groups, metal concentrations were higher in shellfishes (crab, prawn, and oyster) than finfish. Notably, the demersal species *P. laevis, P. trituberculatus* and *E. sinensis* possessed the maximum metal concentrations, while the pelagic species had several degrees of lower metal concentrations. Though most of the species contained the less metal concentrations than national and international guideline values, As concentrations in *C. joyneri* and Cd concentrations in *H. nehereus, L. ocellicauda* and *M. miiuy* exceeded the FAO permissible limit. Seasonally, the metal concentrations varied with both metal and species types. Most of the metals (except Pb) showed a positive relationship, and Cu–Zn, Cu–As and Cu–Cd showed the strongest correlation. All the species possessed bioaccumulative capability, but *P. laevis* and *P. trituberculatus* were highly bioaccumulative. In terms of health risks, both adults and children may experience a high risk for As contamination through seafood consumption. All the fish species asserts non-carcinogenic risks (EDI, THQ and HI) to children, whereas for the adult people *E. sinensis*, *P. laevis* and *P. trituberculatus* consumption would be harmful. However, the CR index values depicted that children are susceptible to carcinogenic risk of AS and Cd contamination, hence unsafe for consumption. Mostly the demersal species of crab, prawn and oyster (especially *P. laevis, P. trituberculatus* and *E. sinensis*) may pose a high risk to the consumer for both carcinogenic and non-carcinogenic risk. Therefore, it is highly recommended to thoroughly examine all the aquatic organisms consumed by human including the metal concentration in various organs. The management authorities and policymakers should take in consideration the current metal and metalloids status in the HB area to provide a healthful environment.

## Supplementary Information

Supplementary Information.

## Data Availability

The datasets generated during and/or analysed during the current study are available from the corresponding author on reasonable request.
