# Publisher Correction: Bioaccumulation and potential human health risks of metals in commercially important fishes and shellfishes from Hangzhou Bay, China

**DOI:** 10.1038/s41598-022-11186-9

**Published:** 2022-04-28

**Authors:** Md Abu Noman, Weihua Feng, Genhai Zhu, M Belal Hossain, Yue Chen, Haifeng Zhang, Jun Sun

**Affiliations:** 1 College of Marine Science and Technology, China University of Geosciences (Wuhan), No.388 Road Rumo, Wuhan, 430074 China; 2 Key Laboratory of Marine Ecosystem Dynamics and Second Institute of Oceanography, Ministry of Natural Resources, Hangzhou, 310012 China; 3 Department of Fisheries and Marine Science, Noakhali Science and Technology University, Sonapur, Noakhali Bangladesh; 4 School of Engineering and Built Environment, Griffith University, Nathan Campus, Griffith, QLD Australia

Correction to: *Scientific Reports* https://doi.org/10.1038/s41598-022-08471-y, published online 17 March 2022

The original version of this Article contained an error in the spelling of the author M Belal Hossain which was incorrectly given as MBelal Hossain. The original Article has been corrected.

