# Supplementary material for: Bioaccumulation and potential human health risks of metals in commercially important fishes and shellfishes from Hangzhou Bay, China
Source: Sci Rep. 2022 Mar 17;12:4634. doi: 10.1038/s41598-022-08471-y (PMC8931090; doi:10.1038/s41598-022-08471-y)
Supplement: Supplementary file 1 — Supplementary Information. [file 41598_2022_8471_MOESM1_ESM.docx]

**Bioaccumulation and Potential Human Health Risks of Metals in Commercially Important Fishes and Shellfishes from Hangzhou Bay, China**

**Md Abu Noman^1^, Weihua Feng^2^, Genhai Zhu^2^, M Belal Hossain^3,4^, Yue Chen^2^, Haifeng Zhang^2^, Jun Sun^1*^**

^1^College of Marine Science and Technology, China University of Geosciences (Wuhan), Wuhan, Hubei 430074, China

^2^Key Laboratory of Marine Ecosystem Dynamics & Second Institute of Oceanography, Ministry of Natural Resources, Hangzhou 310012, China

^3^Department of Fisheries and Marine Science, Noakhali Science and Technology University, Sonapur, Noakhali, Bangladesh.

^4^ School of Engineering and Built Environment, Griffith University, Nathan Campus, QLD, Australia.

**^*^ Corresponding Author**: (phytoplankton@163.com)

Jun SUN, Ph. D

College of Marine Science and Technology

China University of Geosciences (Wuhan)

No.388 Road Rumo, Wuhan, 430074, P.R. China

E-mail: phytoplankton@163.com or sunjun@cug.edu.cn

**Supplementary Figure 1**

Seasonal variation of heavy metal concentration (mg/kg) in *C. nasus*, *C. lucidud*, and *P. laevis.* (Asterisk denotes the level of significance. *p<0.05, **p<0.01, ***p<0.001)


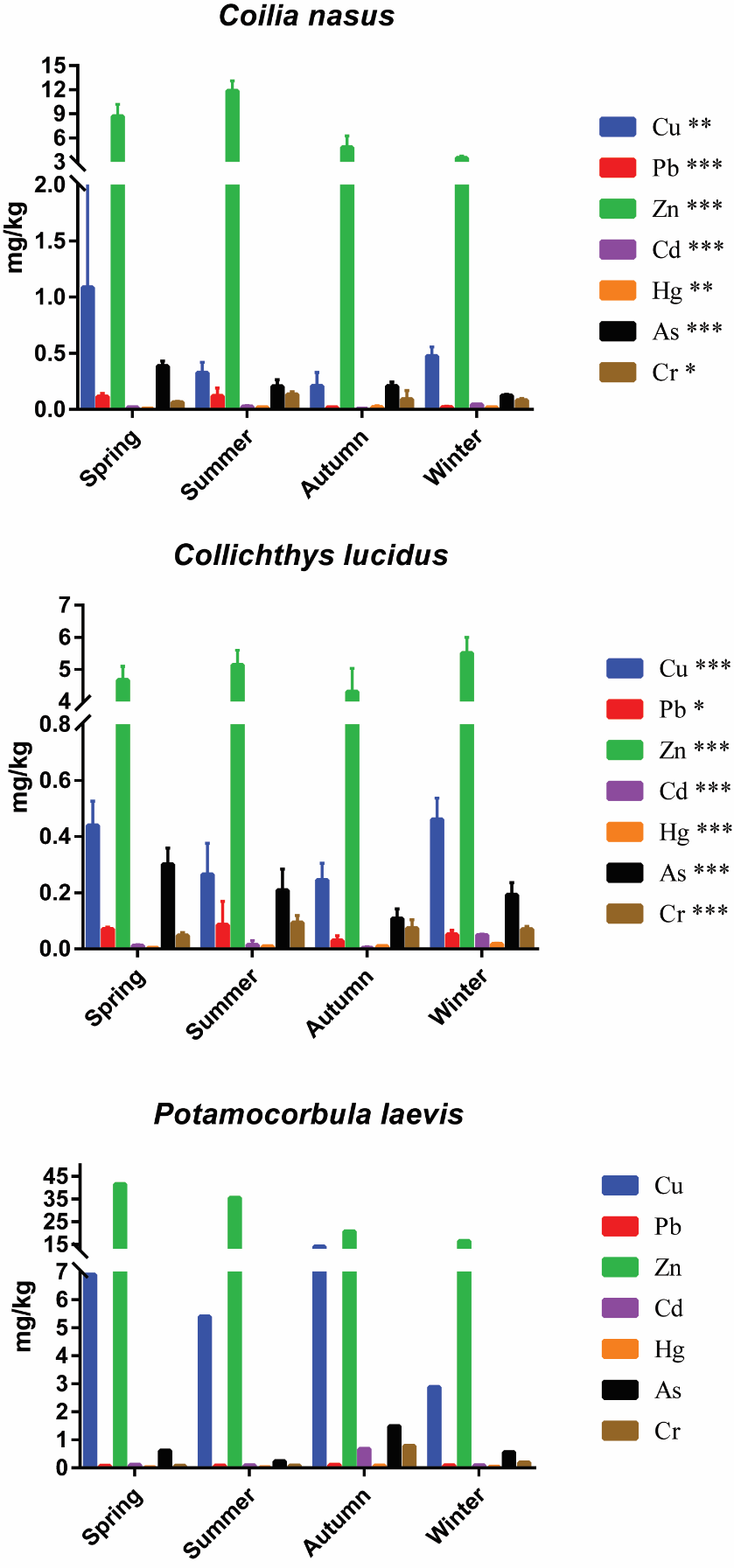


**Supplementary Figure 2**

Pearson’s correlation coefficient matrix of heavy metal in organism’s muscles. (Color bar and circle size denotes the correlation coefficient, P<0.05 are shown in squared-box)


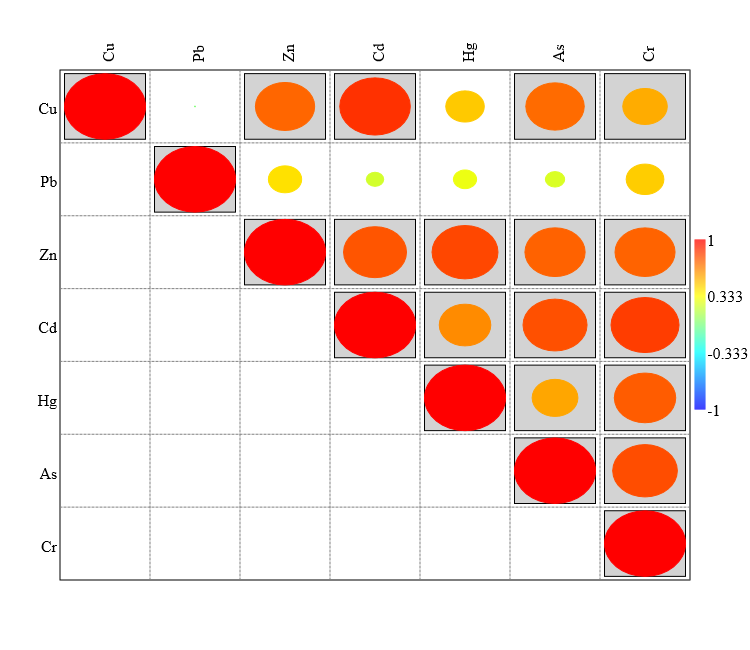


**Supplementary Table 1**

Analytical methods, detection limit and recovery range of heavy metals in organism’s muscle (unit= mg/kg)

| Name | Analysis method name | Analysis Procedure  (National standard) | Detection Limit | Allowable uncertainty | Recovery range |
| --- | --- | --- | --- | --- | --- |
| Cu | Flameless atomic absorption spectrophotometry | 17378.6-2007 / 6.1 | 0.4 × 10^-6^ | ± 0.1 | -0.04 to 0.02 |
| Pb | Flameless atomic absorption spectrophotometry | 17378.6-2007 / 7..1 | 0.04× 10^-6^ | ± 0.07 | -0.05 to 0.04 |
| Zn | Flame atomic absorption spectrophotometry | 17378.6-2007 / 9.1 | 0.4 × 10^-6^ | ± 2.4 | -1.4 to 1.9 |
| Cd | Flameless atomic absorption spectrophotometry | GB 17378.6-2007 / 8.1 | 0.005 × 10^-6^ | ± 0.117 | -0.092 to 0.067 |
| Cr | Flameless atomic absorption spectrophotometry | GB 17378.6-2007 / 10.1 | 0.04× 10^-6^ | ± 0.04 | -0.03 to 0.03 |
| Hg | Atomic fluorescence method | GB 17378.6-2007 / 5.1 | 0.002 × 10^-6^ | ± 0.029 | -0.020 to 0.012 |
| As | Atomic fluorescence method | GB 17378.6-2007/ 11.1 | 0.2× 10^-6^ | ± 3.0 | -2.5 to 0.8 |

**Supplementary Table 2**

Comparison between the estimated daily intake (EDI) and recommended daily allowance (RDA) of Heavy metals for both adult and children. (Values in bold denotes the exceeded permissible limit)

| Metals | Metal concentration | *EDI*  (mg/day/person) | | *RDA*(mg/kg/person) | Reference Dose- *R_f_D*(mg/(kg · day)) |
| --- | --- | --- | --- | --- | --- |
|  | Average ± SD | Adult | Child |  |  |
| Cu | 2.40±2.66 | 1.08 | 4.31 | 35 | 0.04 |
| Pb | 0.05±0.02 | 0.03 | 0.11 | 0.25 | 0.004 |
| Zn | 10.32±7.13 | 4.83 | **19.32** | 11 | 0.3 |
| Cd | 0.07±0.07 | 0.03 | **0.13** | 0.07 | 0.001 |
| Hg | 0.012±0.009 | 0.01 | 0.02 | 0.055 | 0.0005 |
| As | 0.42±0.26 | **0.19** | **0.77** | 0.15 | 0.0003 |
| Cr | 0.11±0.08 | 0.05 | 0.18 | 0.23 | 0.003 |
